# Supplementary material for: Platinum oxide formation under oxygen evolution reaction conditions
Source: Nat Commun. 2026 May 14;17:4368. doi: 10.1038/s41467-026-72954-z (PMC13176324; doi:10.1038/s41467-026-72954-z)
Supplement: Supplementary file 1 — Supplementary Information [file 41467_2026_72954_MOESM1_ESM.pdf]

Supplementary Information for

Platinum Oxide Formation under Oxygen Evolution Reaction Conditions

Leon Jacobse[1,2,*], Ralf Schuster[3], Mona Kohantorabi[1], Silvan Dolling[1,4], Johannes Pfrommer[1,5], Xin Deng[1,3], Tim Weber[5], Olof Gutowski[6], Ann-Christin Dippel[6], Olaf Brummel[3], Yaroslava Lykhach[3], Heshmat Noei[1], Herbert Over[5], Jörg Libuda[3], Vedran Vonk[1], Andreas Stierle[1,4]

[1]Centre for X-ray and Nano Science CXNS, Deutsches Elektronen-Synchrotron DESY, Notkestraße 85, 22607 Hamburg, Germany

[2]Present address: Department of Interface Science, Fritz Haber Institute of the Max Planck Society, Berlin, Germany Notkestraße 85, 22607 Hamburg, Germany

[3]Interface Research and Catalysis, Erlangen Center for Interface Research and Catalysis, Friedrich-Alexander-Universität Erlangen-Nürnberg, Egerlandstraße 3, 91058 Erlangen, Germany

[4]Fachbereich Physik, Universität Hamburg, Jungiusstraße 11, 20355 Hamburg, Germany

[5]Institute of Physical Chemistry and Center for Materials Research, Justus Liebig Universität Gießen, Heinrich-Buff-Ring 17, 35392 Gießen, Germany

[6]Deutsches Elektronen-Synchrotron DESY, Notkestraße 85, 22607 Hamburg, Germany

*email: jacobse@fhi-berlin.mpg.de

1 Electrochemistry

The instrument for combined RDE and HE-SXRD experiments is shown in Fig. S1 and has been described extensively in previous work[1]. Figure S2 shows the Pt(111) voltammetry measured in the RDE-SXRD experiment as well as the steady-state ORR/OER currents. The voltammetry shows the expected features of Pt(111), with some small contributions from step sites, which originates from the edges of the sample as the meniscus was not pushed to its extreme to ensure its stability during the entire experiment. As can be seen in Fig. 2B, the X-ray beam leads to a small (photo)electrochemical current, but this effect is very small and does not affect the reactivity of the sample.

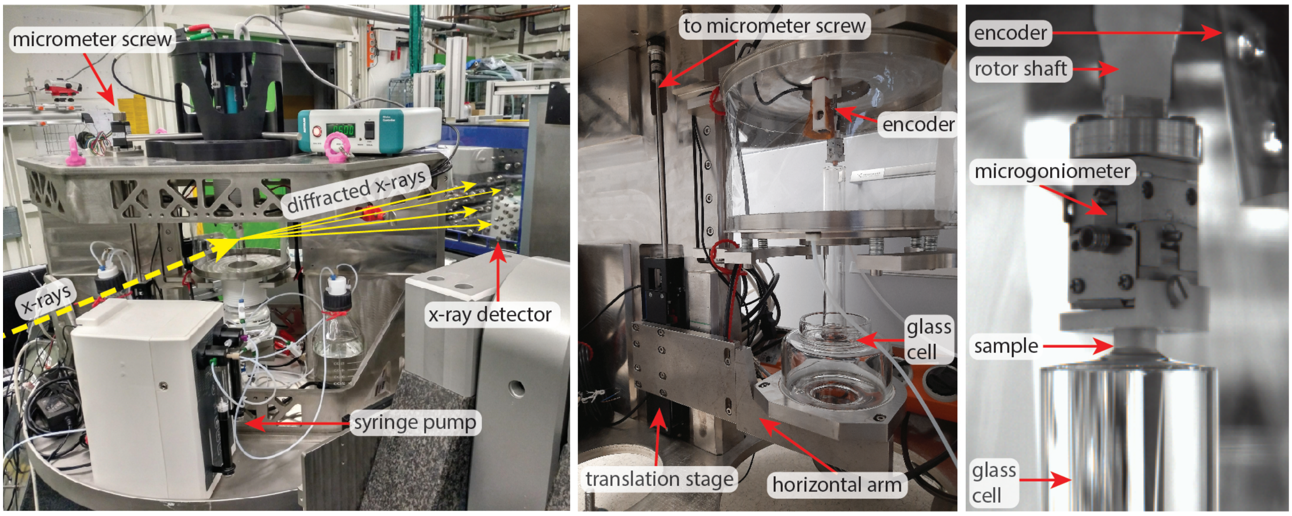

Figure S1: **Experimental setup:** Photographs of the entire setup as mounted at the P07 beamline (left), an overview of the construction to move the cell height (middle), and a zoomed in view on the sample mounting (right). Note that the sample is facing downwards and the diffraction pattern thus appears below the sample surface horizon. Reproduced with permission from Ref.[1]

.

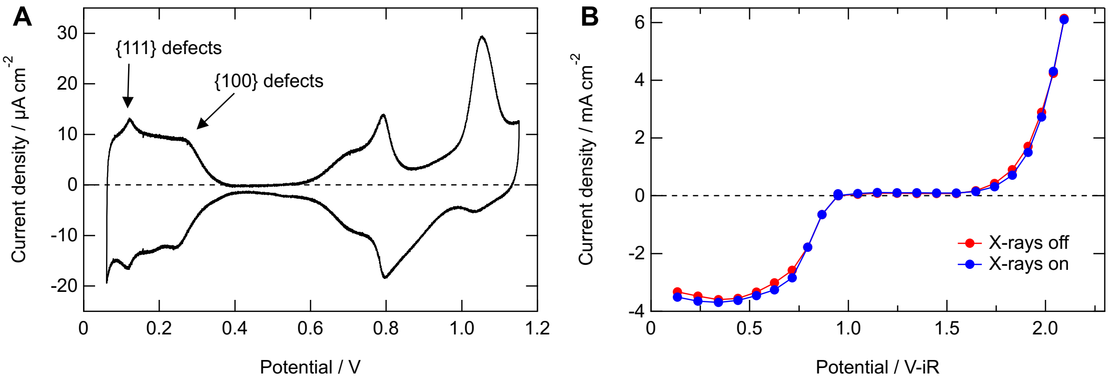

Figure S2: **Electrochemistry:** A) blank CV and B) effect of radiation on ORR/OER. Source data are provided as a Source Data file.

2 Oxidation during ORR

The oxidation of platinum can not only occur electrochemically at large positive potentials, but also chemically by exposing it to a high chemical potential of oxygen (thermal oxidation). Although thermal oxidation experiments are typically performed at much higher temperatures, oxide formation during the oxygen reduction reaction (ORR) is often considered for Pt nanoparticles[2]. However, for macroscale electrodes the voltammetric fingerprint of stripping thermally adsorbed oxygen is quite different from that of the PE process[3]. Furthermore, potentiodynamic HE-SXRD measurements (in a quiescent electrolyte) on Pt(111) did not change upon saturating the electrolyte with O_2[4].
Our RDE-SXRD instrument enables us to better understand this discrepancy by performing full Crystal Truncation Rod (CTR) measurements under constant enhanced mass transport conditions. CTR profiles are, specifically far away from the strong Bragg reflections, highly sensitive to the atomic surface structure. Thus, surface oxidation under ORR conditions would lead to significant changes in the measured signal. Figure S3 shows the (0 1), (0 2), and (1 1) CTRS as a function of potential while the ORR takes place at the electrode surface. Note that, although we did not observe any superstructure signal, the formation of an ordered adlayer of oxygen atoms[5] cannot be excluded as such a signal might be hidden underneath the relatively large background intensity that originates from our hanging meniscus configuration. Experiments using vicinal samples would be beneficial to understand if undercoordinated sites do oxidize during the ORR. However, note that the oxide formation would have to be structurally reversible as voltammetric experiments of such samples do not show changes after performing ORR[6].

3 Operando HE-SXRD

Figure S4 shows the (1 1), (1 0), and (2 0) CTRs as function of potential during an oxidation experiment in the absence of O_2. Like in the ORR experiment, the data below 0.95 V (no vertical offset) overlap. Note that, lead absorber pieces were used to block the high intensity Bragg peaks, leading to gaps in the data at those positions. The continuous RDE rotation across 360 degrees required all Bragg peaks to be blocked, which due to the sample symmetry leads to gaps in the data e.g. at L=1 in the (01) CTR. At higher potentials, the electrode oxidizes similarly to the experiments in the presence of O_2. To visualize this, Fig. S4 also shows the (1 1) CTR data measured in O_2-saturated electrolyte (gray). Only at 1.25 V the structure factors are somewhat different, but it should be noted that at this potential the scattering intensity is very low and these differences could very well be explained by slightly different qualities in the initial surface preparation. The same observation can be made from the comparison of the structure factor at the (1 1 1.5) reflection as shown in Fig. S5. The oscillatory behavior, with intensity minima (i.e. maximum roughness) around 1.25 and 2.09 V alternated with intensities approaching that of the pristine surface in between, is an indication that the oxidation proceeds in a layer-by-layer fashion. This is illustrated by the blue line, which shows the simulated intensity for a perfect layer-by-layer etching process as function of the amount of etched material. The scale of the x-axis is based on the oxide growth rate discussed below. In reality, the oxidation process is more complex and dissolution insignificant[7], which requires an analysis of the entire set of CTR data. Because the data measured in the presence of O_2 has the highest quality and also extend to the highest potential, we use those data for further analysis.
Figure S6 shows reciprocal space maps from (1 0)/(2 0) and (1 1) CTR measurements (A and B, etc. respectively) as a function of potential. No signal due to the formation of an ordered oxide superstructure is observed. The

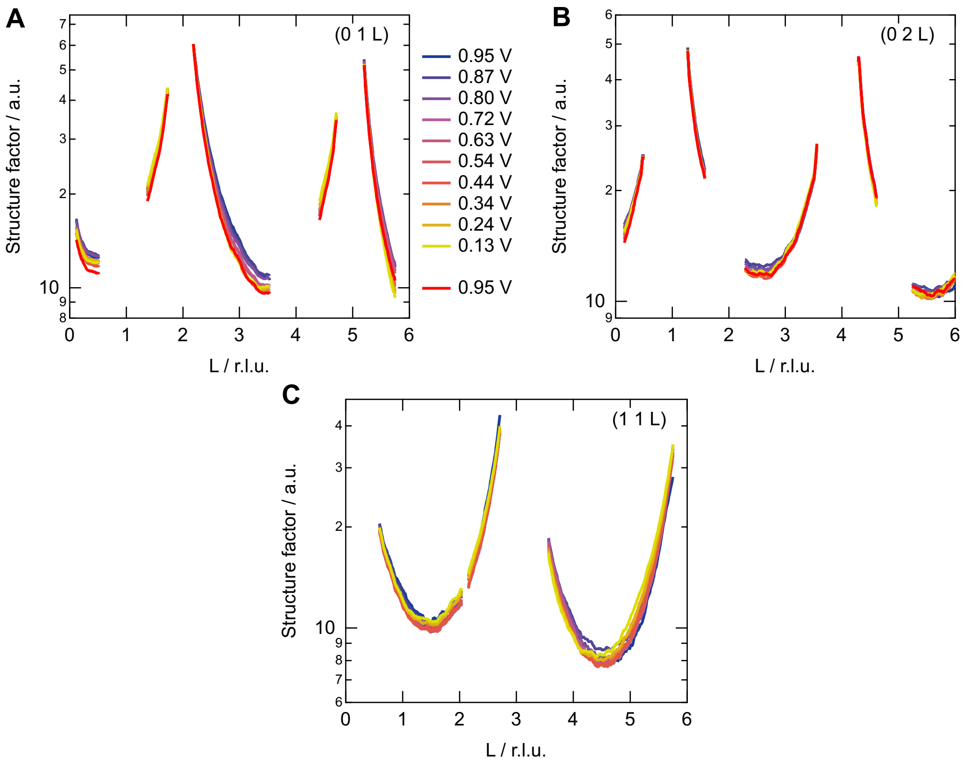

Figure S3: **CTRs during ORR:** evolution of the (0 1), (0 2), and (1 1) CTRs (A-C, respectively) as function of potential during the oxygen reduction reaction measured in O_2-saturated electrolyte. Source data are provided as a Source Data file.

black circular spots are caused by the lead absorber pieces blocking the Bragg peaks. The white lines are the borders of the individual detector modules.

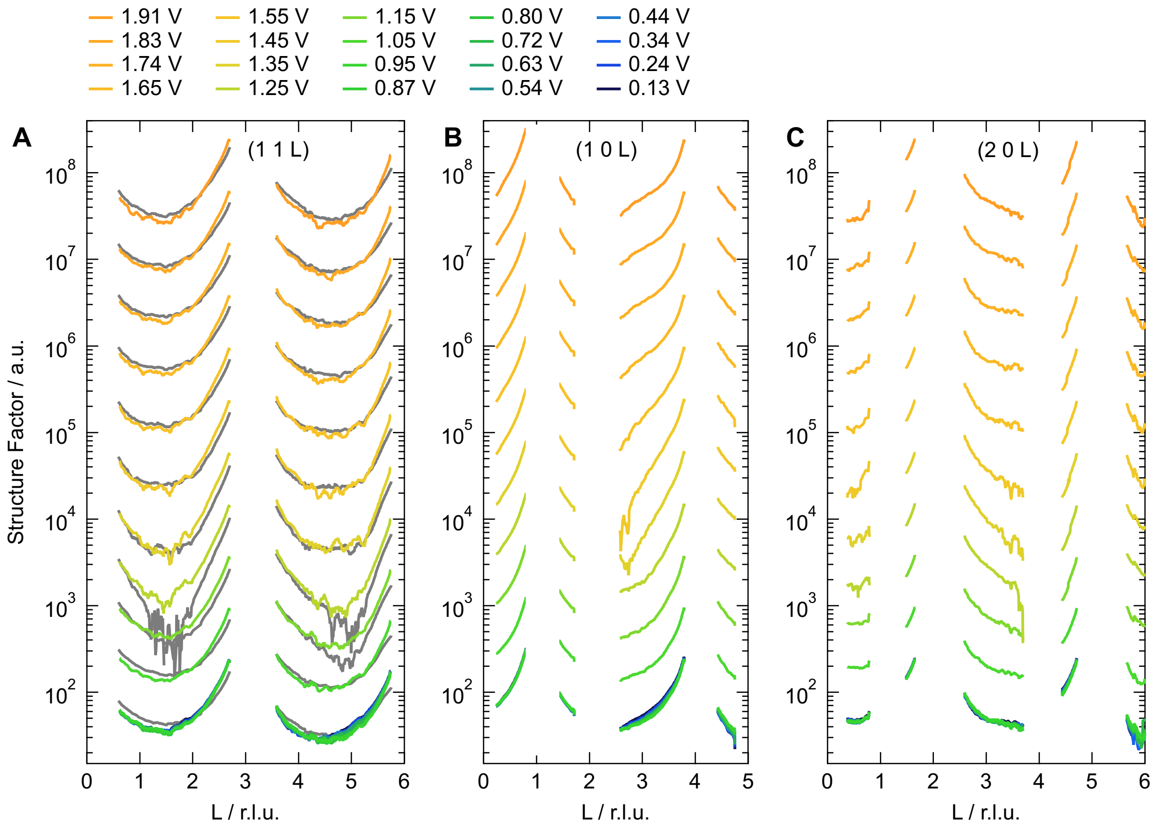

Figure S4: **CTRs in absence of O_2:** (1 1), (1 0), and (2 0) CTRs as function of potential (A-C, respectively). The gray data in (A) are measured in O_2-saturated electrolyte as discussed in the main text. The data above 1 V are vertically offset for clarity. Source data are provided as a Source Data file.

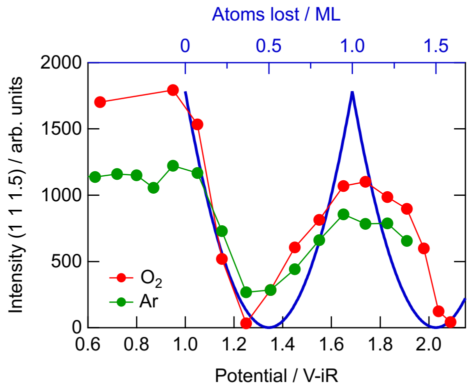

Figure S5: **Intensity at (1 1 1.5):** Evolution of the (1 1 1.5) scattering intensity as function of potential for the experiment performed in O_2- and Ar-saturated electrolyte (red and green, respectively). The oscillation of the signal is a direct (qualitative) indication of the platinum surface being affected in a layer-by-layer mode as is illustrated by the modeled intensity. Source data are provided as a Source Data file.

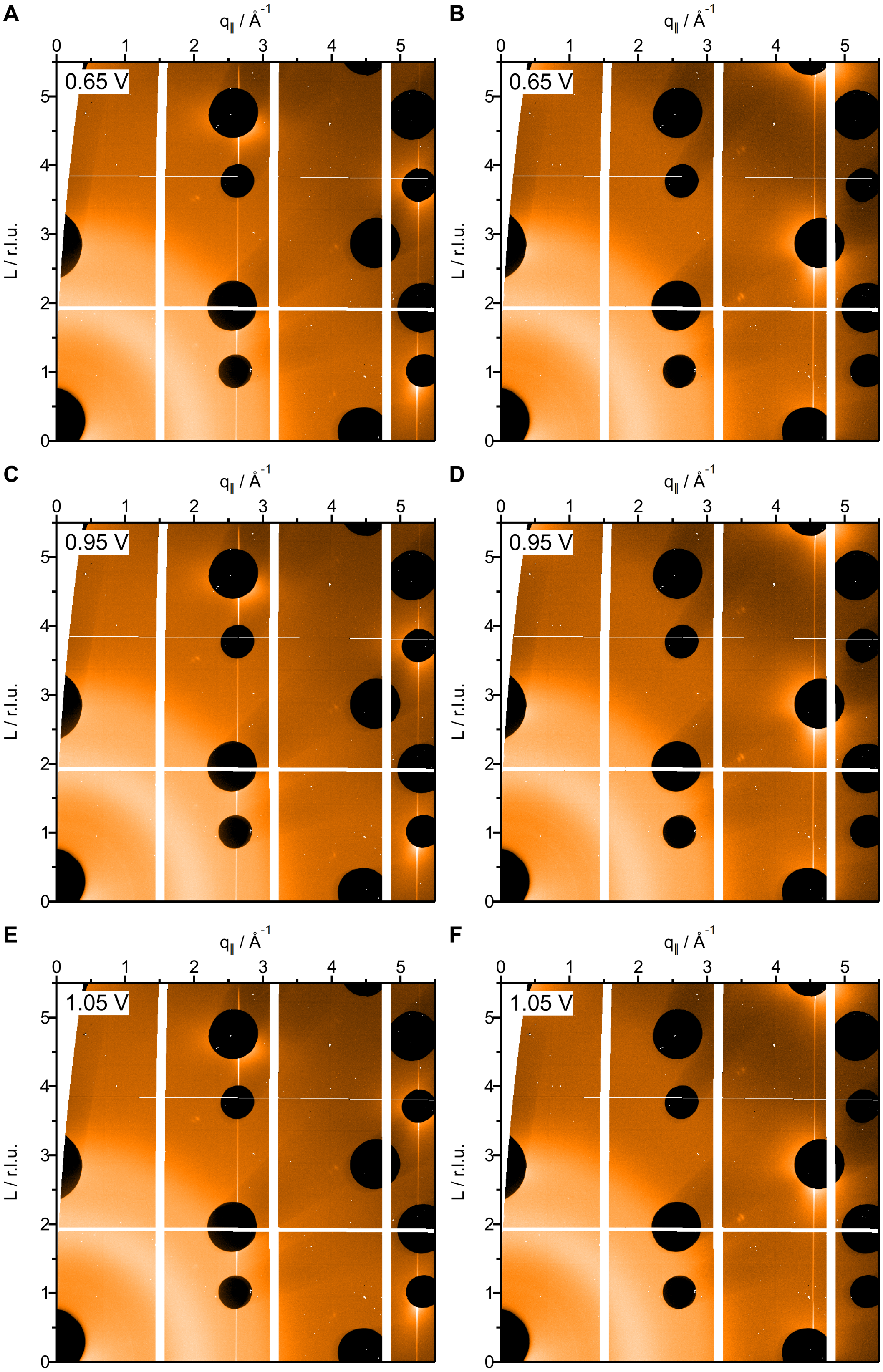

A
B
C
D
E
F
q∥ / Å^-1
L / r.l.u.
0.65 V
0.95 V
1.05 V

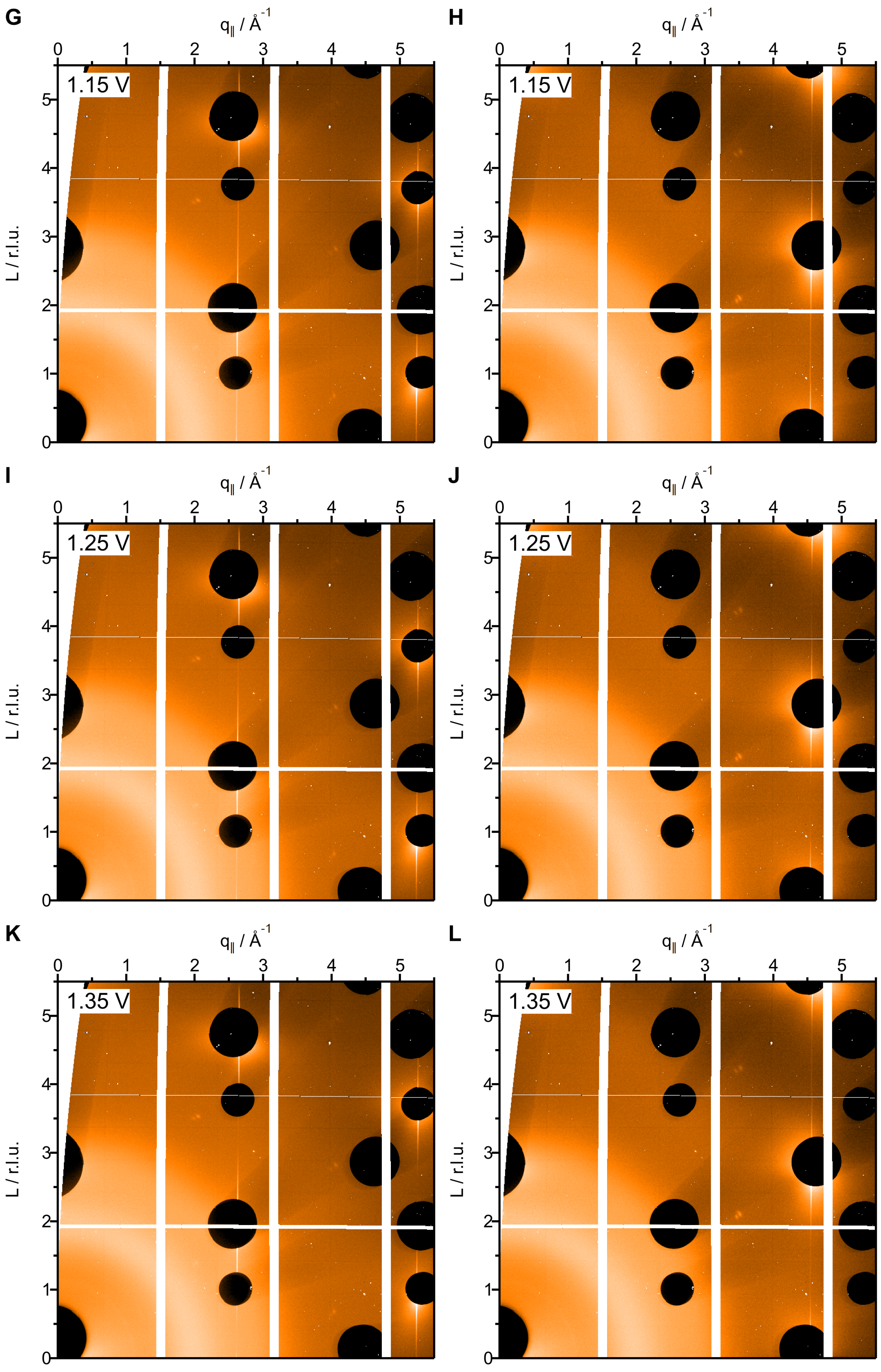
G
H
I
J
K
L
1.15 V
1.25 V
1.35 V
$q_{\parallel}$ / Å^{-1}
L / r.l.u.

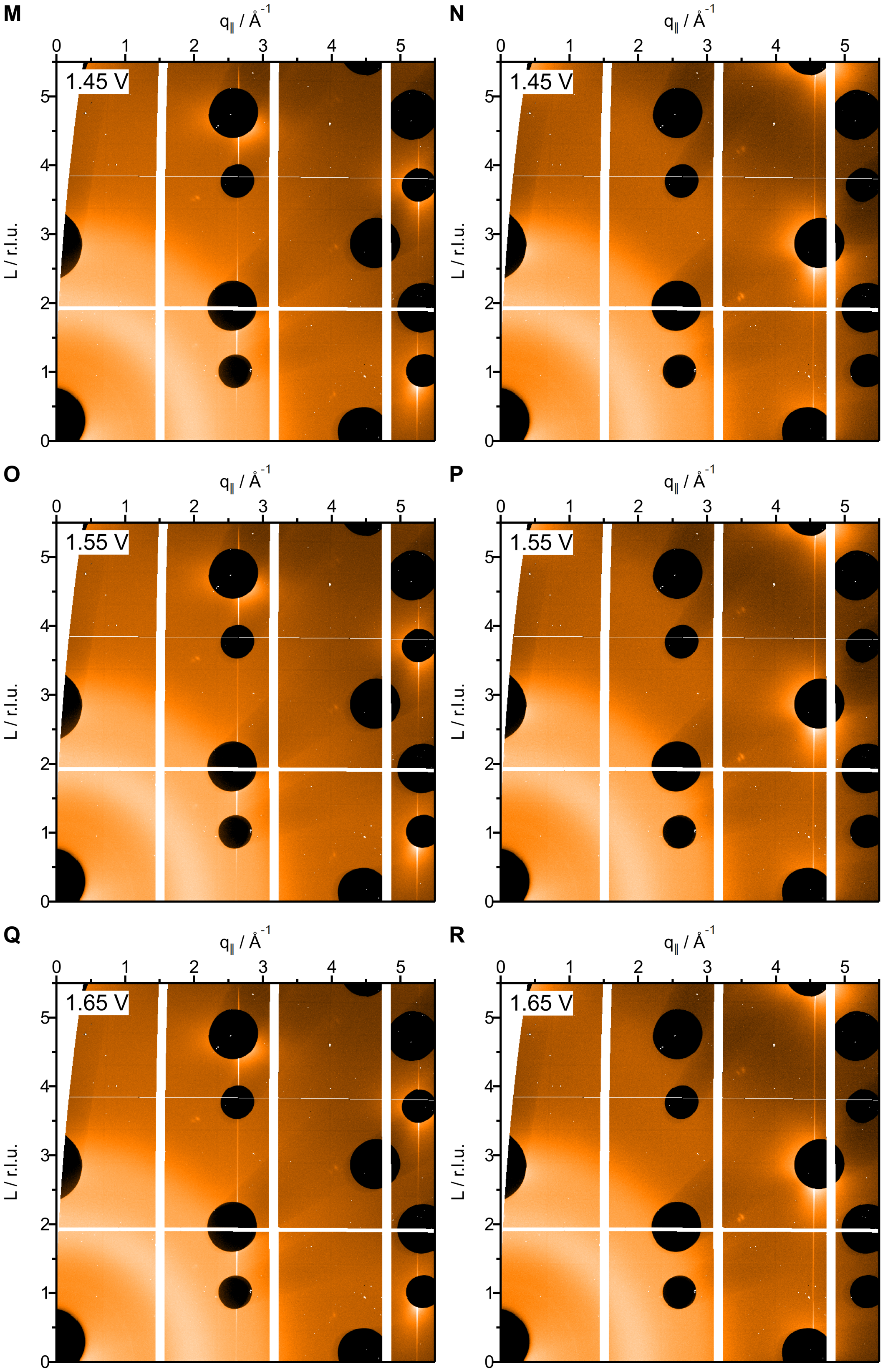
M
N
O
P
Q
R
q|| / Å-1
L / r.l.u.
1.45 V
1.45 V
1.55 V
1.55 V
1.65 V
1.65 V

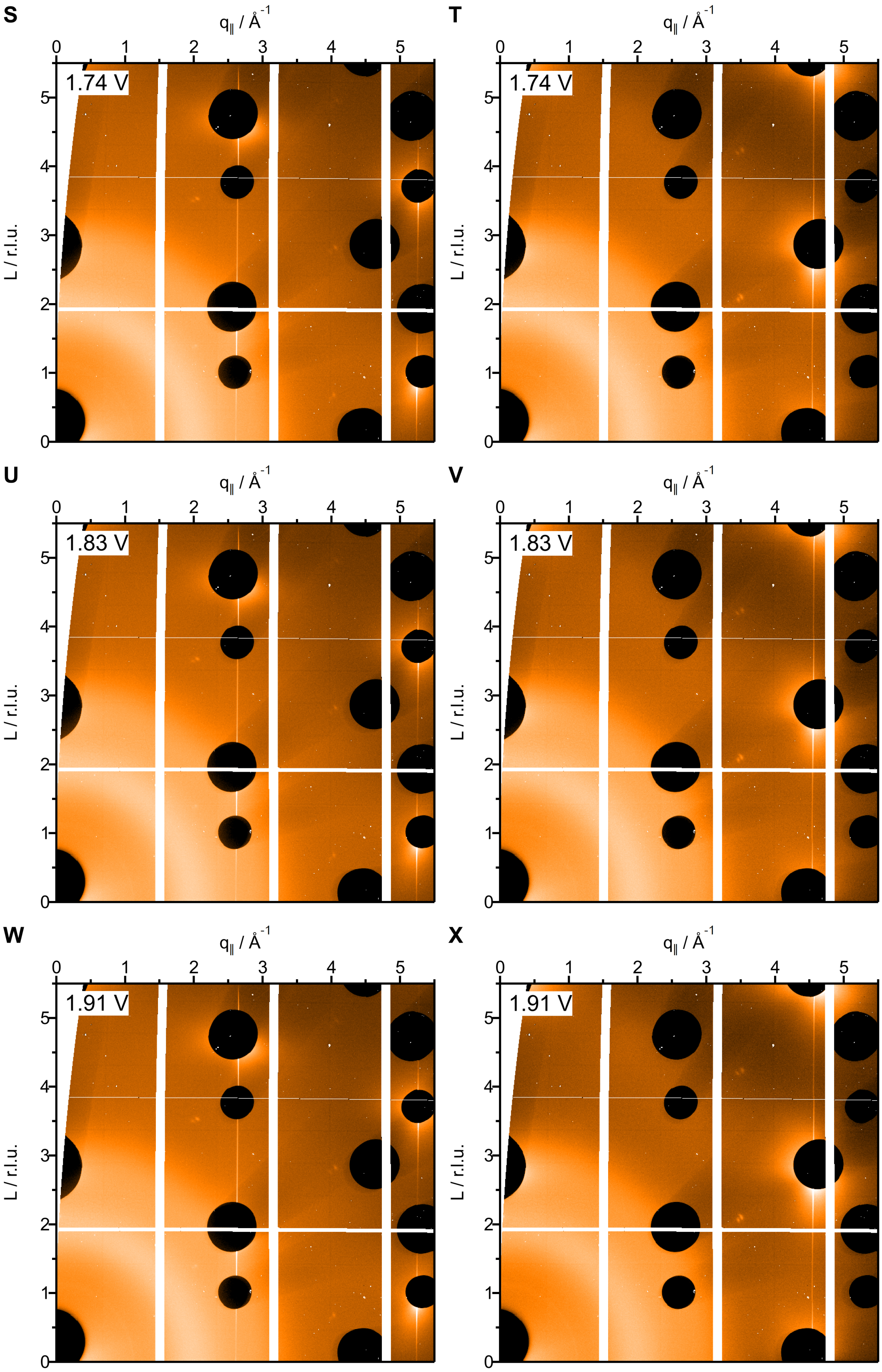
S
T
U
V
W
X
1.74 V
1.83 V
1.91 V
q$_{\|}$ / Å^{-1}
L / r.l.u.

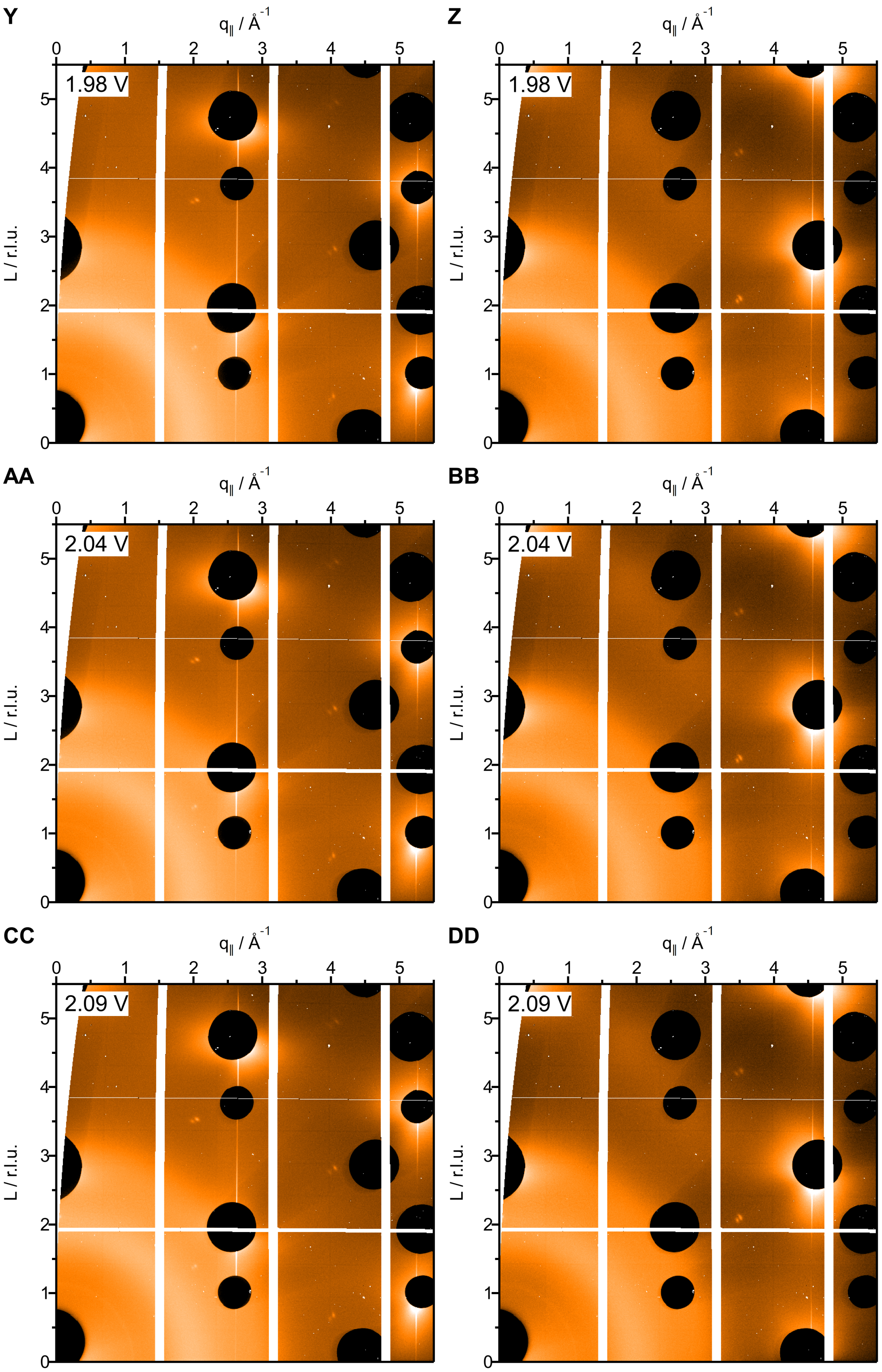

Figure S6: **Reciprocal space maps:** SXRD measurement of the (1 0)/(2 0) and (1 1) CTRs (A and B, etc. respectively) indicating the absence of an ordered oxide superstructure.

4 X-ray Reflectivity

Ex situ X-ray reflectivity (XRR) measurements were used to obtain further information on the disordered oxide surface. Figure S7 shows a continuous $\theta/2\theta$ scan (grey) of the Pt(111) surface after the most oxidizing experiment (leading to the lowest scattering intensity). Rocking curves (red) were used to verify the presence of a significant signal intensity on top of the background. Figure S8 shows the evolution of the different parameter values as determined from the fits of the XRR curves. The density of the water layer was kept constant at its bulk value.

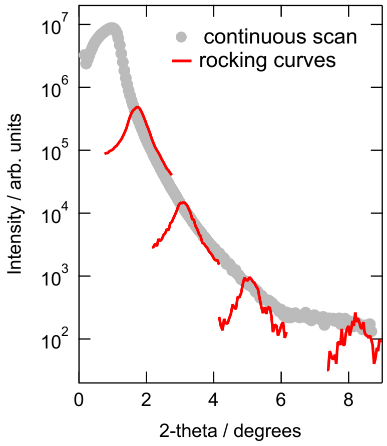

Figure S7: **X-ray reflectivity:** XRR curve (grey) measured in a $\theta/2\theta$ geometry after oxidizing the sample at 2.11 V, together with a selection of the corresponding rocking curves (red). Source data are provided as a Source Data file.

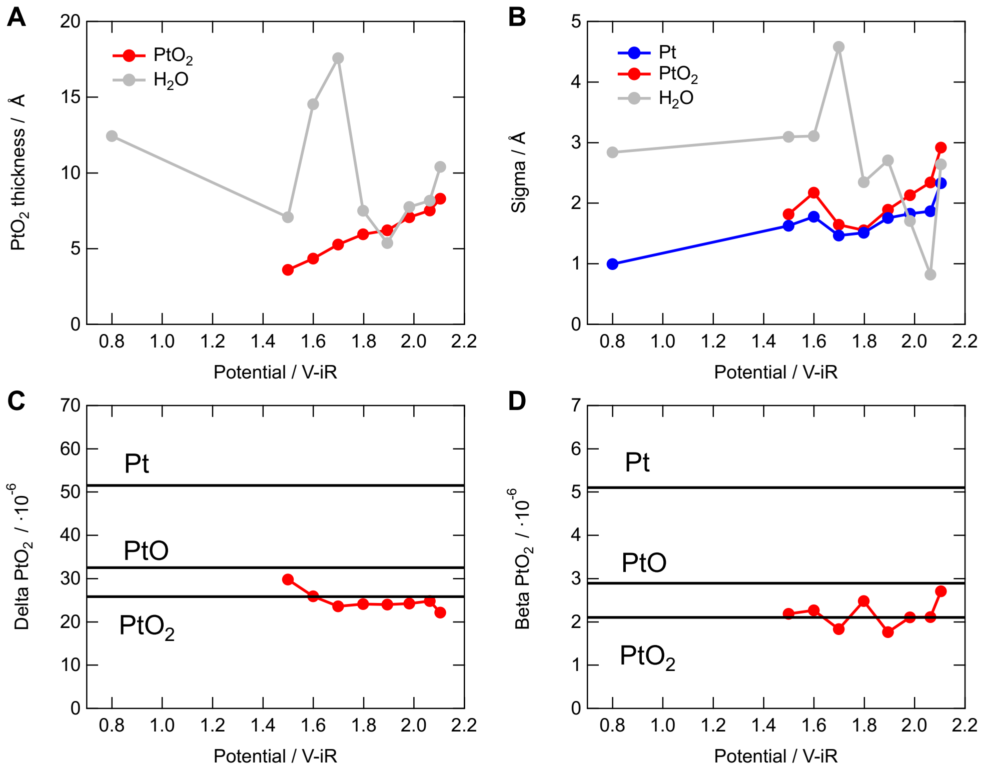

Figure S8: **XRR fit parameter values:** thickness, roughness, real and imaginary refractive index (A-D, respectively) resulting from the XRR analysis. The horizontal lines in C and D indicate the values for bulk Pt, PtO, and PtO_2. Source data are provided as a Source Data file.

5 Electrochemical Impedance Spectroscopy

Figure S9A show EIS data after progressively oxidizing Pt(111) at potentials between 1.3 and 2.11 V. For each experiment the potential was swept with 10 mV·s^{-1} to the upper potential limit, held for 10 minutes and then swept back to 1.3 V. Thus, all spectra were measured at 1.3 V to avoid a contribution from OER. Apart from a very small shift during the first few measurements, which is also seen for repeated measurements at 1.3 V (Fig. S9B), the data overlap perfectly.

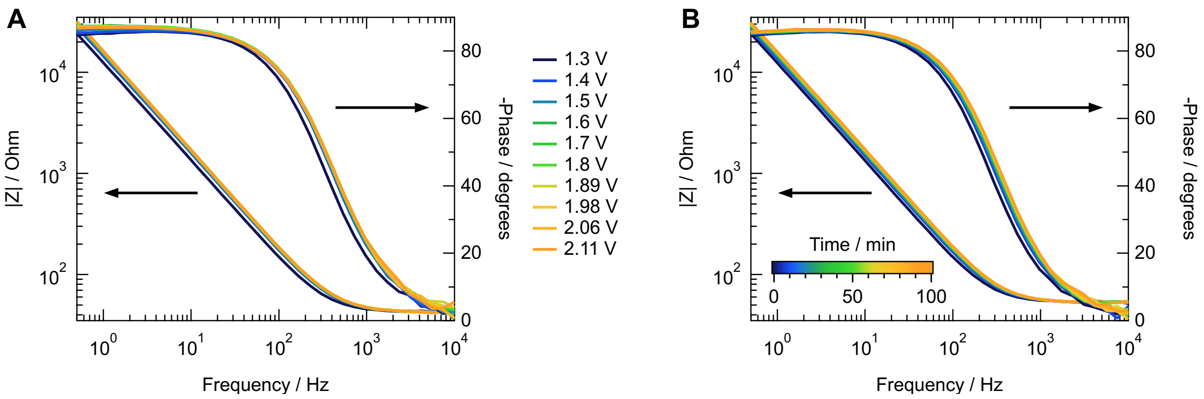

Figure S9: **Impedance spectra:** A) EIS data measured at 1.3 V after oxidizing the sample at potentials between 1.3 and 2.11 V B) EIS data for repeated measurements at 1.3 V. Source data are provided as a Source Data file.

6 X-ray Photoelectron Spectroscopy

Figure S10A shows O 1s spectra, confirming the presence of hydroxide and/or adsorbed water as well as the oxide phase consistent with the interpretation of the Pt 4f assignment. The C 1s spectra (Fig. S10B) are presented to rule out significant contamination role that could influence peak fitting and assignments of the Pt 4f core level. The O 1s peak at 530 eV (red) is attributed to the oxide component, the peak at 531.5 eV (blue) is assigned to hydroxide/H_2O species[8], and the peak at 533 eV is attributed to carboxylic groups, consistent with the observed peaks in the C 1s core level at 286–288 eV. The C 1s peak at 284–284.8 eV is assigned to aliphatic carbon, while the peaks at 286 eV and 288 eV are assigned to C=O and carboxylic species, respectively. Residual carbon species are obviously present due to exposure to air, but we do not expect these to influence the other results. Figure S11 shows the Pt 4f XPS spectra of the pristine surface and after oxidizing the sample at 1.8 V. The latter spectra is measured directly after introducing the sample in UHV, as well as after keeping it inside the system for 72 hours indicating that the oxide is unstable under UHV conditions.

In addition, we performed an independent check to identify the presence of oxidized Pt species on the pristine Pt(111) surface after cleaning by Ar^+ sputtering, in order to compare the sputter-annealed surface with the commonly used flame-annealed preparation. The Pt(111) crystal was cleaned by four cycles of Ar^+ ion sputtering (1.5 kV, 15 min) followed by annealing to 1000 K in UHV for 3 minutes. The surface cleanliness and order were verified by LEED and XPS, as shown in Fig. S12A and S12B, respectively. The asymmetric Lorentzian LF line shape was used for fitting of the Pt 4f spectra (LF(0.9, 2, 20, 50)), which is widely used as a practical asymmetric approximation for metallic Pt surfaces[2]. For both the sputter-annealed surface and the surface after brief exposure to air (2 hours), only sharp metallic Pt 4f signals were observed (Figures S12C and S12D). Using the LF line shape, the FWHM of metallic Pt components was 1.1 eV.

Table S1: **XPS fitting parameters:** Overview of the XPS fitting parameters.

	Pt^0	Pt^{2+}	Pt^{4+}
Line shape	LF(0.9, 2, 20, 50)	GL(30)	GL(30)
Peak position	70.9 74.2 eV	71.9, 75.3 eV	73.8, 77.1 eV
FWHM	0.96-1.1 eV	1.3 eV	1.8 eV

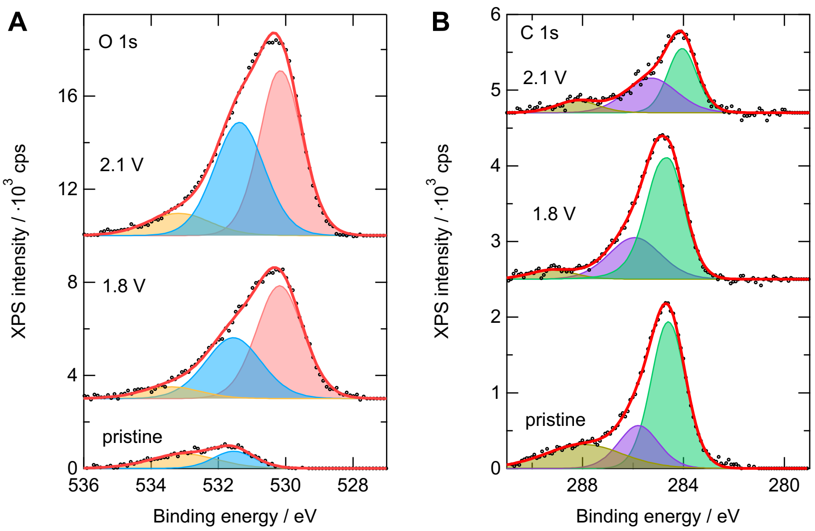

Figure S10: **XPS spectra:** A) O 1s and B) C 1s spectra of the samples shown in Fig. 4 of the main text. Source data are provided as a Source Data file.

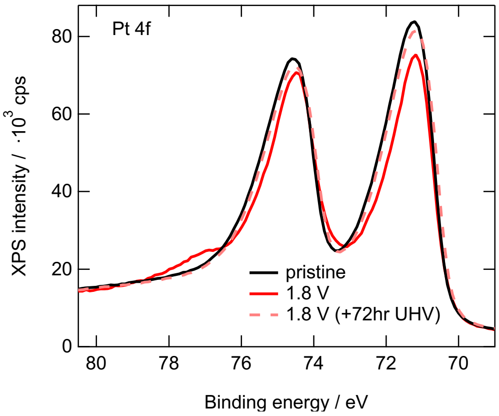

Figure S11: **XPS spectra:** Pt 4f spectra of the pristine surface and after oxidizing it at 1.8 V. Source data are provided as a Source Data file.

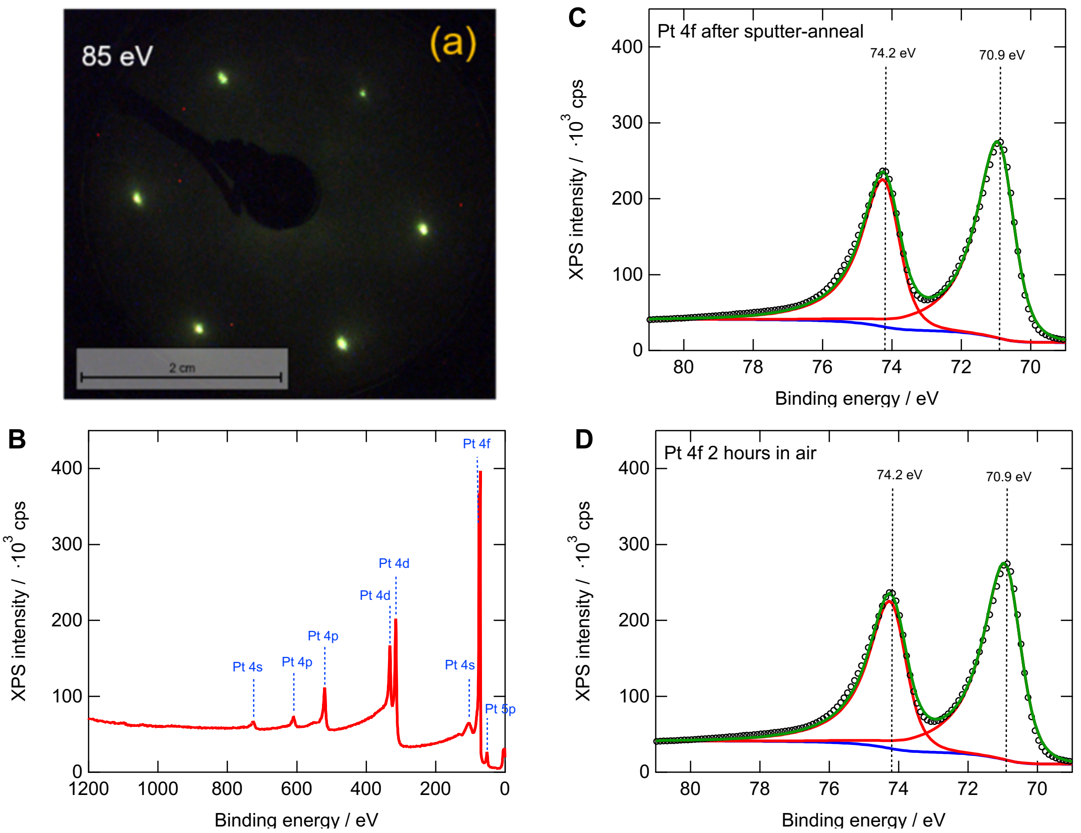

Figure S12: **UHV preparation:** A) LEED pattern at 85 eV and B) XPS survey spectrum of the clean Pt(111) surface after sputter-anneal preparation. Deconvoluted Pt 4f core-level spectra of the sample (C) after sputtering-annealing and (D) after 2 hours of exposure to air. Source data are provided as a Source Data file.

References

[1] Jacobse, L.; Schuster, R.; Pfrommer, J.; Deng, X.; Dolling, S.; Weber, T.; Gutowski, O.; Dippel, A.-C.; Brummel, O.; Lykhach, Y.; Over, H.; Libuda, J.; Vonk, V.; Stierle, A. A combined rotating disk electrode–surface x-ray diffraction setup for surface structure characterization in electrocatalysis. *Rev. Sci. Instrum.* **2022**, *93*, 065111.

[2] Mom, R.; Frevel, L.; Velasco-Vélez, J. J.; Plodinec, M.; Knop-Gericke, A.; Schlögl, R. The Oxidation of Platinum under Wet Conditions Observed by Electrochemical X-ray Photoelectron Spectroscopy. *J. Am. Chem. Soc.* **2019**, *141*, 6537–6544.

[3] Huang, Y. F.; Koper, M. T. Electrochemical Stripping of Atomic Oxygen on Single-Crystalline Platinum: Bridging Gas-Phase and Electrochemical Oxidation. *J. Phys. Chem. Lett.* **2017**, *8*, 1152–1156.

[4] Drnec, J.; Ruge, M.; Reikowski, F.; Rahn, B.; Carlà, F.; Felici, R.; Stettner, J.; Magnussen, O. M.; Harrington, D. A. Pt oxide and oxygen reduction at Pt(111) studied by surface X-ray diffraction. *Electrochem. commun.* **2017**, *84*, 50–52.

[5] Kondo, T. et al. Potential-Dependent Adsorbed Structures of Oxygen Species on Pt(111) Single-Crystal Electrode during Oxygen Reduction Reaction Investigated by In situ Surface X-ray Scattering. 2019; `https://www.ise-online.org/ise-conferences/annmeet/folder/70th_Annual_meeting-BoA.pdf`.

[6] Sugimura, F.; Sakai, N.; Nakamura, T.; Nakamura, M.; Ikeda, K.; Sakai, T.; Hoshi, N. In situ observation of Pt oxides on the low index planes of Pt using surface enhanced Raman spectroscopy. *Phys. Chem. Chem. Phys.* **2017**, *19*, 27570–27579.

[7] Sandbeck, D. J.; Brummel, O.; Mayrhofer, K. J.; Libuda, J.; Katsounaros, I.; Cherevko, S. Dissolution of Platinum Single Crystals in Acidic Medium. *ChemPhysChem* **2019**, *20*, 2997–3003.

[8] Hrbek, T.; Kúš, P.; Rodríguez, M. G.; Matolín, V.; Matolínová, I. Operando X-ray photoelectron spectroscopy cell for water electrolysis: A complete picture of iridium electronic structure during oxygen evolution reaction. *International Journal of Hydrogen Energy* **2024**, *57*, 187–197.
